# Prevalence of Potentially Inappropriate Prescriptions According to the New STOPP/START Criteria in Nursing Homes: A Systematic Review

**DOI:** 10.3390/healthcare11030422

**Published:** 2023-02-01

**Authors:** Isabel Díaz Planelles, Elisabet Navarro-Tapia, Óscar García-Algar, Vicente Andreu-Fernández

**Affiliations:** 1 Faculty of Health Sciences, Valencian International University (VIU), 46002 Valencia, Spain; 2 Neonatology Unit, Hospital Clinic de Barcelona, Grup de Recerca Infancia i Entorn (GRIE), Institut d’Investigacions Biomèdiques August Pi i Sunyer (IDIBAPS), 08036 Barcelona, Spain; 3 Department of Neonatology, Hospital Clínic-Maternitat, ICGON, BCNatal, 08028 Barcelona, Spain

**Keywords:** STOPP, START, nursing homes, potentially inappropriate medications, geriatrics, multimorbidity, polymedication, potentially prescribing omissions, prescribing practice

## Abstract

The demand for long-term care is expected to increase due to the rising life expectancy and the increased prevalence of long-term illnesses. Nursing home residents are at an increased risk of suffering adverse drug events due to inadequate prescriptions. The main objective of this systematic review is to collect and analyze the prevalence of potentially inadequate prescriptions based on the new version of STOPP/START criteria in this specific population. Databases (PubMed, Web of Science and Cochrane) were searched for inappropriate prescription use in nursing homes according to the second version of STOPP/START criteria. The risk of bias was assessed with the STROBE checklist. A total of 35 articles were assessed for eligibility. One hundred and forty nursing homes and more than 6900 residents were evaluated through the analysis of 13 studies of the last eight years. The reviewed literature returned prevalence ranges between 67.8% and 87.7% according to the STOPP criteria, according to START criteria prevalence ranged from 39.5% to 99.7%. The main factors associated with the presence of inappropriate prescriptions were age, comorbidities, and polypharmacy. These data highlight that, although the STOPP/START criteria were initially developed for community-dwelling older adults, its use in nursing homes may be a starting point to help detect more efficiently inappropriate prescriptions in institutionalized patients. We hope that this review will help to draw attention to the need for medication monitoring systems in this vulnerable population.

## 1. Introduction

According to the latest data published by the World Health Organization, by 2050 the population over 60 years of age will represent 22% of the world’s population, having doubled since 2015 [1]. This evolution of the elderly population is mainly due to the increase in life expectancy [2,3], but above all, due to the increase in the survival rate at younger ages, largely due to the evolution and improvement of public health systems. Parallel to all these changes, there has been an evolution in the pattern of disease, and acute and communicable diseases have given way to those of a chronic and degenerative nature [4]. In the aging process, structural and functional changes are produced in the different organs and systems, fundamentally affecting renal, hepatic, cardiac function and the immune system. These physiological alterations will produce changes in the pharmacokinetics and pharmacodynamics of drugs, such as decreased metabolism and excretion of drugs, which will cause changes in the therapeutic effect and increase adverse reactions [2]. These changes that lead to adverse effects and complications, in most cases, are avoidable. In addition, the manifestation of certain diseases can also change, often making diagnosis and treatment more difficult [5].

To all these changes we must add the intrinsic characteristics of the elderly, in most cases polymedicated and with different comorbidities. An average of five diagnoses is estimated in the elderly, regardless of the health care setting of the study [4]. Among the most prevalent diagnoses are pathologies related to the cardiovascular system (CVS), cancer and diabetes [6]. In addition, dementia is one of the most prevalent diseases among people living in nursing homes (NH) [7].

Polypathology in the elderly directly affects the safety of prescribed drugs, increasing the risk of interactions and, therefore, the number of adverse drug reactions (ADRs) [2]. Specifically, older patients residing in NH have comorbidities and a higher average drug consumption than patients residing in the community [8]. One of the factors associated with the occurrence of ADRs in the elderly patient are the potentially inappropriate prescriptions (PIPs) [9]. A drug is considered potentially inappropriate when the risk of its use is greater than the clinical benefit, especially when safer and more effective therapeutic alternatives are available [10]. PIPs also include: (i) the use of medicines at a higher dose than indicated; (ii) the use of medicines with a high risk of drug–drug or drug–disease interactions; (iii) duplications. Not using clinically indicated beneficial medicines are also considered to be PIPs [11].

Due to the complexity of the profile of NH residents, the prevalence of PIPs in these patients is higher than in other studies conducted in other healthcare settings. A systematic review conducted in 2015 by Tommelein et al. [12], conducted in community-dwelling patients, found a PIPs prevalence of 22.6% (CI 19.5–26.7%), almost half the prevalence found in studies conducted in NH residents [13,14]. In addition, a recent systematic review and meta-analysis of observational studies has shown a global prevalence of polypharmacy of 37% among the COVID-19 patients. The authors also showed that the older the COVID patients are, the higher the prevalence of polypharmacy. Therefore, it is possible that in recent years PIPs in NHs have increased even more due to the pandemic [15]

PIPs have a negative impact on older people at multiple levels. A recent meta-analysis has shown a significant association with emergency room visits, adverse drug events, functional decline, health-related quality of life and hospitalizations [16]. Digestive, skin, nervous and CVS are the most affected by the adverse drug reactions [17]. In addition, adherence to treatment is also reduced, and the patient’s physical functionality is diminished. In economic terms, healthcare costs are increased due to longer hospital stays, diagnostic tests and drugs. For example, the total PIPs expenditure in Ireland was found to be almost EUR 46 million, i.e., 9% of the overall expenditure on pharmaceuticals in those aged ≥ 70 years in that country [18]. Given that polypharmacy tend to increase over time [19], it is not excluded that this expenditure is higher. In addition, it also generates a deterioration of trust in the health care systems.

The most widely used explicit methods in recent decades in institutionalized patients to detect PIPs and optimize medicine use have been the Beers’ criteria [20] and Screening Tool of Older Persons potentially inappropriate Prescriptions/Screening Tool to Alert doctors to Right Treatment (STOPP/START) [11,13]. The STOPP/START criteria were first described by Gallagher et al. in 2008 [21], and although not originally intended for use in NH residents, have been shown to be more sensitive than the Beers’ criteria for the detection of PIPs in this patient group [13,22,23]. The STOPP/START criteria were the first European criteria and are currently the most used and validated for elderly people in Europe. Moreover, the STOPP criteria have been used in intervention studies for the deprescription of inappropriate prescriptions to improve adherence and quality of life. Deprescription has also decreased the occurrence of adverse drug reactions [24,25,26]. The new version of the criteria, updated in 2014, is composed of a battery of 115 criteria (81 STOPP criteria and 34 START criteria) that describe the most common prescribing errors and drug omissions in the older adult [27,28]. These new criteria have 28 more indications than the first version and are based on recommendations from clinical studies. These criteria have several advantages over other explicit criteria. On the one hand, the STOPP/START criteria are organized by systems, facilitating their application in daily practice, and on the other hand, they present a list of PIPs by omission (START) criteria.

Since the publication twenty years ago of the first explicit criteria for the detection of PIPs, a high prevalence of PIPs in older people has been demonstrated, regardless of the detection method used and the health care setting where it is applied [7,29,30,31,32,33,34]. Mean prevalence of PIPs of 43% (37.3–49.1%) has even been reported using different screening tools in residents of NH [14]. Other reviews, carried out in the same health care setting with the first version of the STOPP/START criteria, obtained higher prevalence both in the calculation of PIPs by indication and by omission [13,35].

An increasingly aging population, the rise of polymedication, and higher income in NH as the large Baby Boomer generation ages highlights the need to evaluate the new published versions of the explicit criteria in this population. STOPP/START criteria were initially developed for community-dwelling older adults, as the lack of explicit criteria tailored to the NH residents has been an obstacle to assessing the quality of prescribing. To our knowledge, no systematic review of NH residents using the second version of the STOPP/START criteria has been published. Systematic reviews using the latest STOPP/START criteria have been focus on other populations such as older adults living in the community, older lung cancer patients, older adults undergoing surgery or hospitalized older adults [13,36,37,38,39,40,41,42,43]. Existing studies using STOPP/START vs2 are scarce and heterogeneous, making it necessary to compile all the information published to date to summarize and centralize the main results of the use of these new criteria in this specific population. The main objective of this study is to compile and analyze the scientific evidence published to date on the prevalence of PIPs, by indication and by omission, calculated with the new version of the STOPP/START criteria in NH residents. Additionally, the number of PIPs detected and information concerning the main risk factors associated with PIPs in this population will be also discussed. The data presented in this review may help to highlight the need to promote systematic medication reviews in NHs to identify potentially inappropriate prescribing practices and promote medication safety.

## 2. Materials and Methods

### 2.1. Search Strategy

The preferred reporting items for systematic reviews and meta-analysis (PRISMA) statement was the selected guideline to identify, select, evaluate and synthesize the studies for this systematic review. This work was conducted according to the guidelines of 2009 [44], as well as the update of the 2020 PRISMA statement [45]. This review is registered in the PROSPERO database (CRD42022333699).

The research team designed and evaluated the following items: the definition of the research question and objectives; bibliographic search; data collection, evaluation, synthesis and comparison; critical evaluation of the scientific papers selected; and finally, analysis of the main findings and conclusions showing the strengths and weakness of the studies evaluated. The objective of this systematic review is to analyze the prevalence of PIPs based on the second version of the STOPP/START criteria in residents of NH. PubMed (https://pubmed.ncbi.nlm.nih.gov, accessed on 9 September 2021), The Cochrane Central Register of Controlled Trials (https://www.cochranelibrary.com/central, accessed on 19 September 2021), and Web of Science were the electronic databases consulted using the following combination of descriptors and Boolean operators:

PubMed: (Inappropriate prescri* STOPP NURSING HOMES) [Title/Abstract] OR (INAPPROPRIATE PRESCRI* START NURSING HOMES) [Title/Abstract]. For more details about combination of keywords and medical subject heading terms please see Table A1: Detailed PubMed search strategy.

Web of Science: INAPPROPRIATE PRESCRI* STOPP NURSING HOMES (Abstract) OR INAPPROPRIATE PRESCRI* START NURSING HOMES (Abstract) OR INAPPROPRIATE PRESCRI*STOPP RESIDENTS (Abstract) OR INAPPROPRIATE PRESCRI*START RESIDENTS.

The Cochrane Central Register of Controlled Trials: INAPPROPRIATE PRESCRI* STOPP NURSING HOMES [Title/Abstract/Keywords] OR INAPPROPRIATE PRESCRI* START NURSING HOMES [Title/Abstract/Keywords].

### 2.2. Selection Criteria and Data Extraction

Inclusion criteria were original published studies written in English and Spanish published from January 2014 to September 2022, in which the second version of the STOPP/START criteria were used to calculate the prevalence of PIPs in patients residing in NH, regardless of their age. Those studies that calculated the prevalence of PIPs using the second version of the STOPP/START together with other tools were also included if the prevalence was shown separately.

Studies in non-peer-reviewed publications were excluded, as well as book chapters, correspondence, conference abstracts, and reviews. To reduce the risk of biases related to the calculation of a group of criteria related exclusively to a diagnosis or a group of drugs, the studies that calculated only those criteria exclusively related to a diagnosis or a therapeutic group, as well the use of a single criterion for measuring the prevalence of PIPs were also excluded. Similarly, any study that looked at the prevalence of PIPs using the second version of the STOPP/START criteria in a different setting than NH was also discarded of the study.

Once records were identified in the selected time interval from the selected databases (PubMed, Web of Science, and Cochrane), potential articles were collated to Mendeley, and duplicated records removed by I.D.P. The retrieved titles and abstracts were screened independently by I.D.P. and E.N.-T. against the above inclusion and exclusion criteria to identify the potentially relevant studies. In case of inconsistencies between the reviewers about the selected studies, we opted for reconciliation through discussion with another author. The next step was to screen the studies by reading the full text, as sometimes it is not possible to do this by reading the abstract and title alone. This stage was carried out by two researchers from the team.

Prevalence of PIPs was the main variable of interest. To facilitate understanding and analysis of the results, the variables of interest were divided into two groups. The first group included information about the characteristics of the studies and the residents of the NH (age, mean number of drugs prescribed and prevalence of diagnoses) to provide an overview of the selected population. The second group covered the total prevalence results (STOPP/START) and the prevalence of PIPs by indication (STOPP) and by omission (START), separately. In the case of use of more than one screening method or comparing among them, only the results related to our main objectives were considered. Besides prevalence, the number of PIPs detected, and the risk factors related to PIPs according to this second version of STOPP/START criteria were also collected. All the variables compiled in this work and summarized in Table 1 were extracted and tabulated using Microsoft Excel.

### 2.3. Quality Assessment of Studies

The overall quality of the included studies was critically assessed based on the Strengthening the Reporting of Observational Studies in Epidemiology (STROBE) guidelines [46] (by 2 investigators (I.D.P. and E.N.-T.) This checklist contains a total of 22 items, which evaluated the reporting of each study’s title, abstract, introduction, methodology, results, and discussion (Table A2 and Table A3 in Appendix A). Both authors evaluated each of the STROBE items, indicating their presence or absence in the selected studies (see Table A3 in Appendix). The Completeness of Reporting (COR) score for each manuscript was calculated from the formula: COR (%) = (yes ÷ (yes + no) × 100). Quality was measured according to previous studies [47,48], that is, “low” (COR: 0–49%), “moderate” (COR: 50–74%) and “high” if ≥75% of items were met. In the case of any study obtaining a COR of less than 49%, it would be extracted from the review.

If STROBE could not be used because the study was not observational, study quality was assessed using the Grades of Recommendation, Assessment, Development and Evaluation (GRADE) tool. This tool describes four levels of quality: high, moderate, low and very low [49]. The quality of evidence was judged by 2 authors (I.D.P. and E.N.-T.). Disagreements were resolved through a consensus-based discussion. The results of both the STROBE and GRADE quality analysis can be found in Table A4 of the Appendix A.

## 3. Results

**Figure 1:**
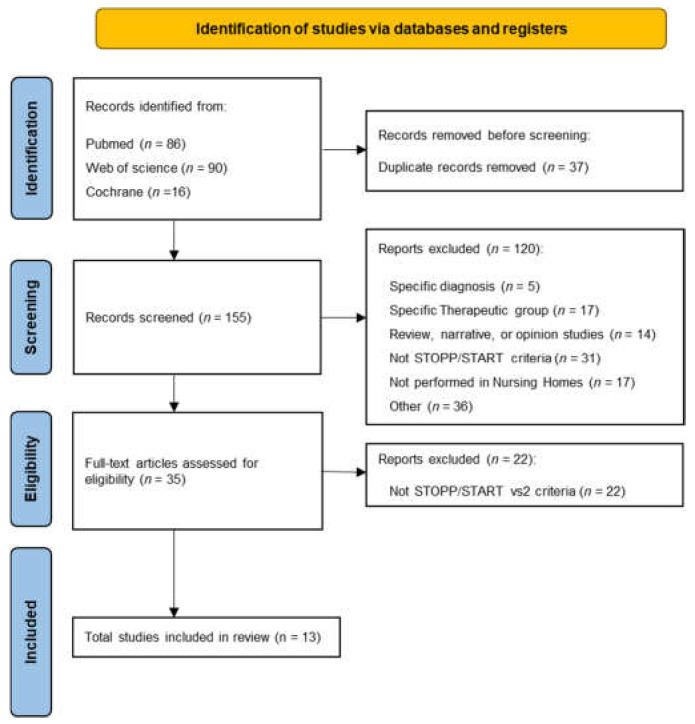
PRISMA flow diagram for the selected studies.

The search of the selected databases (PubMed, Web of Science, and Cochrane) returned 192 unique articles for review in the selected time interval. Once duplicate papers were deleted, the abstract and title of the potentially relevant 155 articles were reviewed, and 120 references were excluded. The reasons for this exclusion were related to the study of populations with a specific diagnosis, inappropriate prescriptions for a specific group of drugs, the non-use of the second version of the STOPP/START criteria, and studies not performed in NH. Finally, 13 studies comprising 140 NH and more than 6900 residents were eligible and included in this systematic review (Figure 1).

### 3.1. Characteristics of the Studies and Residents in NH

The studies were carried out in eight different countries, four in Spain [50,51,52,53], with 2808 residents and 18 NH, two in Portugal [54,55], with 298 residents and 5 NH, and two in Belgium [56,57], whose data were extracted from the same multicenter study (2917 residents in 54 NH). For all other countries: Serbia (400 residents), France (52 residents), Puerto Rico (104 residents), Australia (181 residents), and Malaysia (155 residents) only one study was found [58,59,60,61,62].

In six studies, data were extracted from electronic patient records [50,51,54,55,59,60]. In the other studies, data were collected manually, either by interviewing the patient or reviewing medical reports [52,53,56,57,58,61,62]. As mentioned above, published data from the Belgian studies [56,57] were extracted from the COME-ON study, conducted in 2016 [63]. Regarding the data collection period, eight of the studies obtained data between 2015 and 2018 [50,53,56,57,58,59,61,62]. These data were not available in the other studies.

Most studies included residents over 60 years of age, the average age ranged from 75 [62] to 88 [61]. Only one study excluded those patients aged below 75 years [61]. The results on average drug consumption were variable. Eight of the studies showed drug consumption greater than eight drugs per resident [53,54,56,57,58,59,60,61], four ranged from 6.3 to 7.6 [50,51,52,55], while 3.52 (±3.07) was the lowest consumption [62]. Gutiérrez-Valencia et al. 2018, specified ranges of consumption, with an average drug consumption below 5 in 29 residents and between 6–9 drugs in 81 residents (almost 74% of individuals) [51]. In terms of the number of drugs chronically consumed, the results were also diverse (Table 2). In four studies included, this ranged from 2.69 (±2.49) [62] to 10.1 (±3.2) chronic drugs per resident [61].

Surprisingly, only 53% of the studies analyzed specified the diagnosis of the patients [50,55,57,58,59,62]. Among the most prevalent diseases in these institutionalized patients, diseases related to the CVS stood out, followed by dementias and Alzheimer’s disease. The main characteristics of the studies included, as well as the profile of the residents, are summarized in Table 2.

### 3.2. Prevalence of PIPs According to New STOPP/START Criteria

Of the 13 studies reviewed, only one of them collected both the prevalence of PIPs using STOPP and START in the 233 institutionalized patients. This prevalence reached values of 70.18% [53]. The remaining papers showed the prevalence of PIPs separately, PIP by indication (STOPP) and PIP by omission (START).

Seven studies showed results of prevalence of PIPs STOPP in the population [52,55,58,59,60,61,62]. Although one of them obtained a prevalence of 9.7% of PIPs STOPP [62], surprisingly the prevalence ranged from 67.83% to 87.8% in the remaining six studies.

The absolute values of PIPs STOPP ranged from 1155 to 250. Five studies provided the mean number of PIPs detected per resident, with similar results in four of them [56,59,61,62], ranging from 1.3 to 2, while one of the studies obtained a much higher mean, 10 PIPs STOPP per resident [52].

Only one of the papers reviewed had sufficient information on the residents to be able to calculate all the STOPP criteria described [58]. Other three studies that specified this outcome calculated 29 criteria [54], 62 criteria [61] and 76 criteria [56]. The remaining studies did not specify the number of criteria calculated (Table 3).

The prevalence of PIPs according to START criteria ranged from 39.54% [50] to 99.75% [58]. The absolute value of PIPs calculated in the studies was disparate and ranged from 10 [53] to 2647 [50].

Only four of the thirteen papers showed the mean number of START PIPs per resident, these values ranged from 0.7 to 2 START PIPs per resident [51,56,57,59]. As for the number of START criteria that could be calculated, only four of the reviewed studies specified this [50,54,56,58]. Stojanovic et al. collected sufficient information to be able to calculate all START criteria [58], while in the other three studies the START criteria calculated were 18, 31 and 1 [50,54,56]. All these results are summarized in Table 3.

### 3.3. Factors Associated with the Appearance of PIPs

Information regarding possible factors associated with PIPs is scarce. Only three studies presented results on this subject. One of them associated polypharmacy (OR: 4.81; 95% CI: 2.31–10.0; *p* < 0.001) to the presence of both STOPP and START PPI [62]. However, the other two studies differentiated between factors related to STOPP or START PIPs [56,58]. The main factors associated with the occurrence of STOPP PIPs were age, polypharmacy, and comorbidities. All of these factors, in addition to the dependency ratio, were also associated with the occurrence of PIP START [56,58]. Remarkably, in the study carried out by García-Caballero et al. [52], the types of drugs associated with a greater presence of PIPs were mainly neuroleptics and benzodiazepines (Table 3).

### 3.4. Study Quality Control

The risk of bias is presented for each study in Appendix A (Table A3 and Table A4). The studies included in the article were of moderate to high quality according to the STROBE checklist (see Table A3 in Appendix A) or GRADE tool (in the case of non-observational studies). Half of the articles had a high-quality score (mean COR of 80.2 ± 2.5%), while the other 50% had a moderate COR score (mean COR of 68.8 ± 2.0%).

## 4. Discussion

The STOPP/START criteria were developed two decades ago to detect PIPs and improve therapeutic appropriateness in older patients. However, recent studies concluded that the prevalence of PIPs remains high, especially in NH residents [11,35,64].

Moreover, the literature focused on the STOPP/START criteria published in 2014 to evaluate the efficacy in NH residents are recent and scarce [57,65]. About 50% of NH residents have experienced polypharmacy, while excessive polypharmacy (taking 10 or more drugs) has been observed in 25% of residents [13]. Therefore, the present study performed a systematic review to compile and analyze the scientific evidence published to date on the prevalence of PIPs, by indication and by omission of the second version of the STOPP/START criteria in NH residents.

Perulero et al. calculated a prevalence of 70.18% for PIPs using the second version of these criteria [53], being the first study to obtain it based on the total STOPP/START criteria calculation. However, this percentage was higher than the results found in 2016 by Morin et al. [14], which concluded a prevalence of PIPs ranged from 26.8% in North America to 49% in European countries. Despite both studies being conducted in the same healthcare setting, Morin et al.’s study included different selection criteria (different versions of the Beers’ criteria, STOPP/START criteria vs1 or Laroche’s list of criteria). According to this study, this review also obtained higher prevalences of PIPs in the European population, compared to other countries such as Malaysia. Unfortunately, there are still no studies of PIPs using the new version of STOPP/START to compare with other countries, so future studies in this field are mandatory.

The prevalence of PIPs obtained with other explicit screening criteria as well as in different health care settings were also lower compared with those obtained in the present study [9,12]. This observation again highlights that the use of the second version of the STOPP/START criteria could detect a higher number of PIPs in NH residents. Although the lack of studies in larger populations requires us to take this statement with caution, the present study demonstrates an alarming level of PIPs in Spain [50,51,52]. The reason could be due to the multidose dispensing system, which exposes the patient to a higher risk of PIPs, the need to integrate pharmacists in NH or the drug dispensing practice itself. Recent data indicated 78.8% and 96.8% of polymedication and inappropriate medications of a NH located in the province of Leon (Spain) [66]. These results highlight the importance of using updated criteria to detect PIPs and the need for periodic evaluation of prescriptions in these vulnerable patients.

Cardiovascular diseases and specifically arterial hypertension were the most prevalent diagnoses observed. This conclusion is consistent with a recently published study by Mills et al. [67], which indicated that more than a quarter of the world’s adult population suffers from hypertension. Dementia was also the second most prevalent pathologies, according to other studies carried out in similar health care setting [7,59,68,69].

Regarding the age of the residents, the application of this inclusion criterion showed heterogeneity due to some studies including all residents [50,52,59] and others including those aged 65 years or older [51,53,54,55,56,57,58,60]. Only one study was especially restrictive, including patients beyond 75 years [61]. Although the mean age remained constant between studies (around 80 years), this could add bias in the study.

The present review shows a relatively homogeneous prevalence between 67.8% and 87.8% of STOPP PIPs, in contrast to the low prevalence of 9.7% obtained by Liew et al. [62]. Nevertheless, Liew’s study also showed low polymedication in the population analyzed. This result is in the same vain with Storms et al. [13], that included several methods of PIPs detection and observed lower prevalence of PIPs as average drug consumption decreased.

PIPs prevalence for the same health care setting showed remarkable differences between the first version of STOPP and the second performed in this review. The studies by Storms et al. and Hill-Taylor et al. [13,35] showed prevalence of STOPP PIPs ranging from 23.7–79% (median 61.1%) to 62.4–70.5%, respectively, lower than the prevalence presented in this study. Authors also obtained a higher prevalence with the STOPP criteria than with the 2003 Beers criteria [7]. These results are consistent with previous studies [22,70,71,72].

It also observed a high prevalence of START PIPs (39.6–99.7%), compared to the values observed in previous reviews using the first version of the criteria [13]. It is important to note that high prevalence has not only been found in patients residing in NH. Tommelein et al. found in community-dwelling older people across Europe that about one in five older patients in Europe was exposed to PIPs [12]. These data were obtained with the previous version of the STOPP/START criteria, where some implicit criteria had not been implemented. The authors wondered whether the use of these new criteria would generate higher prevalence ratios indeed. It is necessary for more studies to corroborate this point, but we can hypothesize, based on the results obtained in this review, that the update from the first to the second version of the STOPP/START criteria produced substantial differences in the calculation of PIPs prevalence.

Unfortunately, similar to the STOPP criteria, only a few studies reported the number of START criteria calculated from the medical records of the residents or the face-to-face interviews [50,56,58]. The higher the number of START criteria calculated, the higher the prevalence obtained. Therefore, the prevalence of PIPs START can be compared among studies if the number of START criteria calculated is reported.

Regarding the factors associated with PIPs, several authors associated polypharmacy with an increase in the PIPs occurrence [4,18,31,73]. However, this concurrence with the conclusions obtained in this review contrasts with the results obtained by other authors, which do not establish an association between the consumption of drugs and the presence of PIPs [7,65]. Tommelein et al. reviewed polypharmacy as a risk factor in 52 manuscripts from 23 different countries, obtaining a strong positive association with PIPs [12]. Importantly, the authors also found that poor economic situation and low functional status were also positively associated with PIPs. However, these data were not usually considered in the included studies.

In addition to polypharmacy, age also seems to be associated with a high prevalence of PIPs. These results are in concordance with Storms et al. [13], although their study was conducted in residential long-term care facilities. The presence of comorbidities is another of the factors apparently related to the appearance of PIP, but we cannot state this with certainty because only seven of the total included studies provided data on diagnoses. If we focus on PIPs by omission calculated with the START criteria, the dependency index (calculated with the Katz index) would also be a risk component associated with PIPs. These results agree with the findings of Renom-Guiteras et al. [74], in a study conducted in Europe with elderly people with dementia. They found that the higher the dependency in activities of daily living, the higher the number of PIPs.

In concordance with previous findings, we observed that neuroleptics and benzodiazepines were the most common drugs associated with a greater number of PIPs. Although antihistamines do not usually appear in such studies and represents a minor percentage, it is important to highlight that they are also associated with PIPs, as previously mentioned Tommelein et al. in a previous study [12]. The use of nonsteroidal anti-inflammatory drugs (NSAIDs) is widespread in this population, estimating that 15% of the people in residential aged care use an NSAID long term [75]. In addition, NSAIDs are some of the most frequent PIPs for both STOPP/START and Beers criteria [23]. Therefore, it is necessary to pay special attention to the prescription of this type of drugs, due to NSAIDs has been prescribed by duplicate in the same claim, together with opiates [18].

The updated version of the STOPP/START criteria was published in 2014, so the literature found in institutionalized patients is still scarce. Furthermore, it is necessary for the application of these criteria not only prescription and diagnostic data, but also additional clinical data of the patients. This study has demonstrated a lack of information in the studies both in the electronic and manual records, which prevents the calculation of all the criteria required in the new version of STOPP/START. However, this limitation is common in similar studies to the current review [13,14]. In addition, only a few articles detailed the number of criteria calculated, which made the comparison between studies and the generalization of results observed complex.

The heterogeneity of studies, the lack of uniformity in the criteria measured, and the few studies conducted to date with these criteria in this specific population have made it impossible to conduct a meta-analysis without bias to estimate the prevalence of PIPs in NH residents.

The use of PIPs detection criteria, such as STOPP/START, has been shown to have a positive impact on the reduction of PIPs [25,73]. Furthermore, the systematic review conducted by Wright et al. [76] shows that the benefits of applying PIPs detection criteria in daily clinical practice also entail associated benefits such as a reduction in polymedication, ADRs and healthcare costs. In addition to these new criteria, we would also like to present other suggestions that, together with the new STOPP/START criteria, would help to further reduce PIPs. The integration of pharmacists in NHs to implement pharmacotherapy recommendations, as well as the analysis of medication at the time of admission by a pharmacist in cooperation with the patient’s physician would help to establish individualized pharmacotherapy monitoring during the stay in the center.

To date, this is the first systematic review that analyzes PIPs in institutionalized patients according to the latest version of the STOPP/START criteria. Despite limitations, findings of this review suggest an awareness of the importance of monitoring inappropriate medication use in this vulnerable population.

## 5. Conclusions

This systematic review shows that the use of the second version of STOPP/START criteria still reports high prevalence of PIPs in nursing homes, as in the first version. Although direct comparative studies are needed, we have obtained higher prevalence values in some studies compared to other different studies using the first version. However, direct comparison studies are mandatory to demonstrate this observation. This study also highlights a high prevalence of polypharmacy and comorbidities in these adults. The dependency in activities of daily living could be also a risk factor for PIPs. The increase in life expectancy generates a paradigm shift that leads to a change in the health needs of the entire population. There is still little evidence on the use of this version of the STOPP/START criteria in NH residents. More studies should be carried out using explicit methods for detecting PIPs, to unite criteria among health professionals. This would help to optimize and adapt medication in institutionalized older adults. The adaptation of health systems and the development of new tools to reduce adverse reactions and improve the quality of life of our elders is one of the greatest challenges we face as a society.

## Figures and Tables

**Table 1:** Variables obtained in the selected studies.

Characteristics of the Studies	Results Related to PIPs
Author (year; country)	Prevalence of PIPs according to STOPP/START criteria
Residents (% of women)	Number of STOPP criteria calculated
Number of NH	Number of PIPs detected according to STOPP criteria
Data collection method	Prevalence of PIPs according to STOPP criteria
Study design	Average PIPs detected according to STOPP
Inclusion criteria	Number of START criteria calculated
Patient age	Number of PIPs detected according START criteria
Number of drugs prescribed	Average PIPs detected according to START
Most prevalent diagnoses	Prevalence of PIPs according to START criteria
	Risk factors associated with PIPs

Abbreviations: PIPs: Potentially Inappropriate Prescriptions; NH: Nursing Homes.

**Table 2:** Summary of the main characteristics of the studies and nursing home residents included in this study.

Author (Year)/ Country	Residents *n* (% Women)	NH (*n*)	Data Collection Method (Period of Study)	Study Design	Inclusion Criteria	Age Average (SD/Range)	Average No. of Drugs	Diagnosis *n* (%)	Quality Score
Carvalho et al. (2019)/ Portugal [54]	208 (68.75%)	4	Electronic records (NS)	Descriptive study cross-sectional	>65 y	87 (10)	8 (5)	NS	High
Stojanovic et al. (2020)/ Serbia [58]	400 (69%)	1	Review of medical records at the patient’s first visit (January-June 2018)	Retrospective observational study	>65 y At least 1 chronic prescription drug	83 (11)	8 (5)	Arterial hypertension: 358 (89.5) Angina pectoris: 181 (45.2) Dementia: 151 (37.7) Depression: 135 (33.7) Psychosis: 133 (33.2) Sleep disorders: 124 (31) Heart failure: 105 (26.2) COPD: 68 (17) Infarction: 631 (15.7) Anxiety: 57 (14.2) Osteoporosis: 45 (11.2)	High
Anrys et al. (2018)/ Belgium [56]	1410 (72%)	54	Data extracted from the COME-ON multicenter study (April 2015-June 2016)	Cross-sectional descriptive study	≥65 y Patients not in palliative care	87 (82–91)	9 (6–12)	NS	High
Liew et al. (2019)/ Malaysia [62]	155 (44.5%)	4	Data collected manually by patient interview (November–December 2016)	Cross-sectional multicenter study	≥60 y At least 1 prescribed drug **Exclusion:** residents unable to sign informed consent form	75 (8.49)	**Total drugs:** 3.52 (3.07) **Chronic drugs:** 2.69 (2.49)	Cardiovascular disease: 102 (65.8) Endocrine disease: 56 (36.1) Respiratory disease: 17 (11) Gastrointestinal disease: 15 (9.7)	High
Gaubert et al. (2019)/ France [59]	52 (83%)	1	Electronic records (January–March 2015)	Prospective observational study	All residents of the socio-health center	84 (9)	8.5 (3.5)	Depression: 37 (71) Dementia: 33 (63) Chronic constipation: 33 (63) Hypertension: 29 (56) Osteoporosis: 18 (35) Osteoarthritis: 12 (23)	High
Díaz et al. (2021)/ Spain [50]	2251 (69%)	13	Electronic records (2016–2018)	Retrospective observational descriptive study	All residents of the socio-health center	79.5 (78.3–80.4)	**Total drugs:** 6.30 (6.0–6.4) **Chronic drugs:** 4.5 (4.4–4.7)	Alzheimer’s disease: NS Gastroesophageal reflux: NS Severe anxiety: NS Cerebral vascular disease: NS COPD: NS Chronic atrial fibrillation: NS	High
Nieves-Pérez et al. (2018)/ Puerto Rico [60]	104 (72%)	3	Electronic records (NS)	Cross-sectional descriptive study	≥65 y At least 1 prescribed drug 1 or more chronic diseases and data in the electronic medical record	84 (7.67)	8.6 (3.41)	NS	Moderate
Monteiro et al. (2020)/ Portugal [55]	90 (78.9%)	1	Electronic records (NS)	Cross-sectional descriptive study	≥65 y	84 (65–103)	7.6 (NS) **<5 drugs:** 26 rs **5–9 drugs:** 30 rs ≥**10 drugs:** 33 rs	Diseases of the cardiovascular system: 72 (80) Endocrine and metabolic system diseases: 46 (51) Mental disease: 43 (47.8) Diseases of the musculoskeletal system: 32 (35.5)	Moderate
Gutiérrez-Valencia et al. (2018)/ Spain [51]	110 (71.8%)	2	Data obtained from electronic records, subsequently anonymized, encoded and stored for further analysis (NS)	Cross-sectional cohort study	≥65 y	86.3 (7.3)	NS **5–9 drugs:** 81 rs <**5 drugs:** 29 rs	NS	Moderate
García-Caballero et al. (2018)/ Spain [52]	115 (61.74%)	1	Data collected manually and subsequently entered into an Excel created to detect PIP (NS)	Feasibility study	All residents of the socio-health center	79 (11.44; 46–102)	6.77 (2.92)	NS	Moderate
Perulero et al. (2016)/ Spain [53]	332 (NS)	2	Individualized information was collected for each patient (March–May 2015)	Prospective observational study	≥65 y	83.9 (7.6)	8.7 (4) ≥**10 drugs:** 39.5% rs	NS	Moderate
Strauven et al. (2019)/Belgium [57]	1507 **Intervention group:** 791 (69.9%) **Control group:** 716 (73.4%)	54	Data from a web site created for data collection and filled in by the study investigators (Intervention period: May 2015 to June 2016)	Randomized blinded study (multicenter).	≥65 y Patients without palliative care	**Intervention group:** 87 (82–91) **Control group:** 87 (83–91)	**Intervention group:** 9 (6–12) **Control group:** 9 (6–11)	**Intervention group:** Hypertension (56) Dementia (59.2) Osteoarthritis (63.3) **Control group:** Hypertension (56.1) Dementia (54.2) Osteoarthritis (66.2)	Moderate
Eshetie et al. (2020)/ Australia [61]	181 (54.7%)	NS	Manually collected data (June–July 2017)	Prospective multicenter observational study	≥ 75 y ≥5 drugs prescribed prior to admission to the hospital	**With dementia:** 88.4 (83–92) **Without dementia:** 87 (82–91)	**ADMISSION:** **With dementia** Total drugs: 9.5 (3.5) Chronic drugs: 8.8 (3.2) **Without Dementia** Total drugs: 11 (3.4) Chronic Drugs: 10 (3.2)	**ADMISSION:** Pneumonia/lower respiratory tract infection: 45 (24.9) Falls: 25 (13.8) Cardiovascular problems: 21 (11.6)	Moderate

Abbreviations: COPD: Chronic obstructive pulmonary disease; NH: nursing homes; NS: not specified; PIPs: potentially inappropriate prescriptions; rs: residents; SD: standard deviation; y: years.

**Table 3:** Summary of the main results of potentially inappropriate prescriptions in the resident population of NH.

Author (Year)/ Country	Prevalence of PIPs According to STOPP/ START Criteria	No. of Criteria Calculated STOPP	PIPs Detected According to STOPP Criteria	Average PIPs Detected According to STOPP	Prevalence of PIPs (STOPP Criteria)	No. of Criteria Calculated START	PIPs Detected According to START Criteria	Average PIPs Detected According to START	Prevalence of PIPs (START Criteria)	Risk Factors Associated with PIPs
	*n* (%)	(*n*)	*n* (%)	Mean (SD; Range)	*n* (%)	(*n*)	*n* (%)	Mean (SD; Range)	*n* (%)	
Carvalho et al. (2019)/ Portugal [54]	NS	29	529 (32.5) **Most prevalent criteria:** STOPP K1: 134 STOPP K2: 99	NS	NS	1	NS	NS	NS	NS
Stojanovic et al. (2020)/ Serbia [58]	NS	All	841 (NS) **Most prevalent section:** STOPP K: 448 (53.1) STOPP D: 357 (42.3)	NS	344 (86) **Most prevalent criteria:** STOPP K1: 253 (NS) STOPP D5: 207 (NS) Neuroleptics: 152 (NS) STOPP D6: 100 (NS)	All	1067 (NS) **Most prevalent section:** START I: 627 (52.4) START A: 318 (26.5)	NS	399 (99.7) **Most prevalent** **criteria:** START I1: 399 (NS) START I2: 228 (NS) START A3: 99 (NS)	**STOPP** Age (*ρ* = 0.17; *p* = 0.02) Prescribed drugs (*ρ* = 0.17; *p* = 0.003) **START** Age (*ρ* = 0.10; *p* = 0.02) Prescribed drugs (*ρ* = 0.17; *p* = 0.0005) Number of diagnoses (*ρ* = 0.40; *p* < 0.0001) CCI (*ρ* = 0.31; *p* ≤ 0.0001) MCI (*ρ* = 0.35; *p* < 0.0001)
Anrys et al. (2018)/ Belgium [56]	NS	76	NS **Most prevalent criteria:** STOPP K1:659 (46.7) STOPP D5: 644 (45.7) STOPP K2: 417 (29.6) STOPP I1: 190 (13.5) STOPP D9: 184 (13.0)	2 (NS)	NS	31	NS **Most prevalent criteria:** START E5: 726 (51.5) START A3: 303 (21.5) START E4: 295 (20.9) START G3: 221 (15.7) START A6:196 (13.9) START E3: 191 (13.5)	2 (NS)	1199 (85)	**STOPP** Number of drugs: 5–9 (RR = 2.29; CI: 1.23–2.75); *p* < 0.01 ≥10 (RR = 4.27; CI: 3.60–5.11); *p* < 0.01 Comorbidities **START** Age: >85 (RR = 1.21; CI: 1.02–1.44); *p* = 0.029 Comorbidity: CIRS g ≥ 17 (RR = 1.81; CI: 1.58–2.06); *p* < 0.01 Dependence: Katz Index ≥ 20 (RR = 1.34; CI: 1.16–1.54); *p* < 0.01
Liew et al. (2019)/ Malaysia [62]	NS	NS	NS **Most prevalent criteria:** STOPP D: 8 (40) STOPP J: 4 (20) STOPP F: 4 (20)	1.23 (0.44)	16 (9.7)	NS	NS	NS	NS	Polypharmacy (OR: 4.81; CI 95%: 2.31–10) *p* < 0.001
Gaubert et al. (2019)/ France [59]	NS	NS	NS	2 (1.4; 0–6)	45 (86.5) **Most prevalent criteria:** STOPP A2: 33 (63) STOPP A1: 26 (50) STOPP A3: 18 (35)	NS	NS	0.7 (0.6; 0–2)	30 (57.7) **Most prevalent criteria:** START E5: 28 (54) START A4: 3 (6)	NS
Díaz et al. (2021)/ Spain [50]	NS	NS	NS	NS	NS	18	2647 (NS)	NS	1765 (39.54) **Most prevalent** **criteria**: START E2: NS (94.4) START E7: NS (87.5) START H2: NS(88.6) START A5: NS(84.0) START A6: NS(89.6)	NS
Nieves-Pérez et al. (2018)/ Puerto Rico [60]	NS	NS	417 (NS)	NS	91 (87.5) **Most prevalent criteria:** STOPP A1: 82 (NS) STOPP K1: 42 (NS) STOPP D5: 41 (NS) STOPP D9: 27 (NS) STOPP K2: 26 (NS) STOPP A3: 17 (NS)	NS	162 (NS)	NS	89 (85.58) **Most prevalent criteria:** START A3: 53 (NS) START E5: 49 (NS) START A5: 14 (NS)	NS
Monteiro et al. (2020)/ Portugal [55]	NS	NS	250 (NS)	NS	77 (85.5) **Most prevalent criteria:** STOPP A2: 58 (NS) STOPP D5: 54 (NS) STOPP K1: 54 (NS) STOPP K2: 28 (NS) STOPP A3: 12 (NS)	NS	68 (NS)	NS	52 (57.7) **Most prevalent** **criteria**: START I1: 36 (NS) START E4 and A3: 8 (NS)	NS
Gutiérrez-Valencia et al. (2018)/ Spain [51]	NS	NS	NS	NS	NS	NS	NS	**Frail participants:** 1.9 (NS) **Non-frail participants:** 1 (NS)	**Frail participants:** NS (87.5) **Non-frail participants**: NS (50) OR: 7.00 (CI 95%: 1.3–36.6) **Most prevalent criteria:** START E4: 26 (23.6) START E3: 21 (19.1) START A6: 10 (9.1) START A8: 10 (9.1)	NS
García-Caballero et al. (2018)/ Spain [52]	NS	NS	1155 (NS)	10 (NS)	NS (67.83)	NS	NS	NS	NS	**Drugs associated with a greater number of PIP:** Neuroleptics: 41.48% Benzodiazepines: 16.48% diuretics: 10.80% anticholinergics: 7.95% antihistamines: 5.68
Perulero et al. (2016)/ Spain [53]	233 (70.18)	NS	NS	NS	NS **Most prevalent criteria:** STOPP A1: 111 (29.2) STOPP D5: 110 (28.9) STOPP A2: 46 (21.1) STOPP C1: 35 (9.2)	NS	10 (NS)	NS	NS	NS
Strauven et al. (2019)/ Belgium [57]	NS	NS	NS	NS	NS **Most prevalent criteria in intervention group:** STOPP K1: NS (54.3) STOPP D5: NS (53.9) STOPP K2: NS (37.2) STOPP I1: NS (14.5) STOPP D9: NS (12.9) **Most prevalent criteria in control group:** STOPP K1: NS (55.9) STOPP D5: NS (53.6) STOPP K2: NS (33.5) STOPP I1: NS (12.9) STOPP D9: NS (16.6)	NS	NS	**Intervention group:** 2 (1–3) **Control group:** 2 (1–3)	NS **Most prevalent criteria in intervention group:** START E5: NS (48.9) START A3: NS (14.1) START G3: NS (20.7) START E4: NS (27.2) START E3: NS (18.5) **Most prevalent criteria in control group:** START E5: NS (52.9) START A3: NS (21.9) START G3: NS (20.9) START E4: NS (19.8) START E3: NS (12.8)	NS
Eshetie et al. (2020)/ Australia [61]	NS	62	NS	**Dementia:** 2 (1–4) **Non-dementia:** 2 (1–4)	**Dementia:** 71 (78) **Most prevalent criteria in dementia group:** Use of drugs with anticholinergic properties: 32 (35.2) STOPP F2: 29 (31.9) STOPP K1: 16 (17.6) STOPP A3: 14 (15.4) STOPP B7: 13 (14.3) STOPP K2: 13 (14.3) **Non-dementia** 79 (87.8) **Most prevalent criteria in non-dementia group:** Use of drugs with anticholinergic properties: 22 (24.4) STOPP F2: 43 (47.8) STOPP B7: 23 (25.6) STOPP D5: 22 (24.4) STOPP L3: 16 (17.8)	NS	NS	NS	NS	NS

Abbreviations: CCI: Charlson Comorbidity Index; CI: Confidence Intervals; CIRS-G: Cumulative Illness Rating Scale for Geriatrics; MCI: Medicine comorbidity index; NS: not specified; OR: odds ratio; *p*: *p*-value; PIPs: potentially inappropriate prescriptions; RR: relative risk; ρ: Spearman ρ correlation coefficient; SD: standard deviation.

## Data Availability

Not applicable.

## Abbreviations

ADRs	Adverse drug reactions
CVS	Cardiovascular system
GRADE	Grading of Recommendations, Assessment, Development and Evaluation
NH	Nursing homes
NSAIDs	Nonsteroidal anti-inflammatory drugs
PIPs	Potentially inadequate prescriptions
PRISMA	Preferred reporting items for systematic reviews and meta-analysis
START	Screening Tool to Alert to Right Treatment
STOPP	Screening Tool of Older Persons’ Prescriptions

## Appendix A

**Table A1:** Detailed PubMed search strategy (PubMed).

((((“inappropriate”[All Fields] OR “inappropriately”[All Fields] OR “inappropriateness”[All Fields]) AND “prescri*”[All Fields] AND (“potentially inappropriate medication list”[MeSH Terms] OR (“potentially”[All Fields] AND “inappropriate”[All Fields] AND “medication”[All Fields] AND “list”[All Fields]) OR “potentially inappropriate medication list”[All Fields] OR “stopp”[All Fields])) AND “nursing homes”[Title/Abstract]) OR (((“inappropriate”[All Fields] OR “inappropriately”[All Fields] OR “inappropriateness”[All Fields]) AND “prescri*”[All Fields] AND (“start”[All Fields] OR “started”[All Fields] OR “starting”[All Fields] OR “starts”[All Fields])) AND “nursing homes”[Title/Abstract]))
Translations Inappropriate: “inappropriate”[All Fields] OR “inappropriately”[All Fields] OR “inappropriateness”[All Fields] STOPP: “potentially inappropriate medication list”[MeSH Terms] OR (“potentially”[All Fields] AND “inappropriate”[All Fields] AND “medication”[All Fields] AND “list”[All Fields]) OR “potentially inappropriate medication list”[All Fields] OR “stopp”[All Fields] INAPPROPRIATE: “inappropriate”[All Fields] OR “inappropriately”[All Fields] OR “inappropriateness”[All Fields] START: “start”[All Fields] OR “started”[All Fields] OR “starting”[All Fields] OR “starts”[All Fields]

**Table A2:** STROBE checklist for observational studies.

	Item No	Recommendation
**Title and abstract**	1	(*a*) Indicate the study’s design with a commonly used term in the title or the abstract
		(*b*) Provide in the abstract an informative and balanced summary of what was done and what was found
**Introduction**		
Background/rationale	2	Explain the scientific background and rationale for the investigation being reported
Objectives	3	State specific objectives, including any prespecified hypotheses
**Methods**		
Study design	4	Present key elements of study design early in the paper
Setting	5	Describe the setting, locations, and relevant dates, including periods of recruitment, exposure, follow-up, and data collection
Participants	6	(*a*) *Cohort study*—Give the eligibility criteria, and the sources and methods of selection of participants. Describe methods of follow-up *Case-control study*—Give the eligibility criteria, and the sources and methods of case ascertainment and control selection. Give the rationale for the choice of cases and controls *Cross-sectional study*—Give the eligibility criteria, and the sources and methods of selection of participants
		(*b*) *Cohort study*—For matched studies, give matching criteria and number of exposed and unexposed *Case-control study*—For matched studies, give matching criteria and the number of controls per case
Variables	7	Clearly define all outcomes, exposures, predictors, potential confounders, and effect modifiers. Give diagnostic criteria, if applicable
Data sources/ measurement	8	For each variable of interest, give sources of data and details of methods of assessment (measurement). Describe comparability of assessment methods if there is more than one group
Bias	9	Describe any efforts to address potential sources of bias
Study size	10	Explain how the study size was arrived at
Quantitative variables	11	Explain how quantitative variables were handled in the analyses. If applicable, describe which groupings were chosen and why
Statistical methods	12	(*a*) Describe all statistical methods, including those used to control for confounding
		(*b*) Describe any methods used to examine subgroups and interactions
		(*c*) Explain how missing data were addressed
		(*d*) *Cohort study*—If applicable, explain how loss to follow-up was addressed *Case-control study*—If applicable, explain how matching of cases and controls was addressed *Cross-sectional study*—If applicable, describe analytical methods taking account of sampling strategy
		(*e*) Describe any sensitivity analyses
**Results**		
Participants	13	(a) Report numbers of individuals at each stage of study—e.g., numbers potentially eligible, examined for eligibility, confirmed eligible, included in the study, completing follow-up, and analysed
		(b) Give reasons for non-participation at each stage
		(c) Consider use of a flow diagram
Descriptive data	14	(a) Give characteristics of study participants (e.g., demographic, clinical, social) and information on exposures and potential confounders
		(b) Indicate number of participants with missing data for each variable of interest
		(c) *Cohort study*—Summarise follow-up time (e.g., average and total amount)
Outcome data	15	*Cohort study*—Report numbers of outcome events or summary measures over time
		*Case-control study—*Report numbers in each exposure category, or summary measures of exposure
		*Cross-sectional study—*Report numbers of outcome events or summary measures
Main results	16	(*a*) Give unadjusted estimates and, if applicable, confounder-adjusted estimates and their precision (e.g., 95% confidence interval). Make clear which confounders were adjusted for and why they were included
		(*b*) Report category boundaries when continuous variables were categorized
		(*c*) If relevant, consider translating estimates of relative risk into absolute risk for a meaningful time period
Other analyses	17	Report other analyses done—e.g., analyses of subgroups and interactions, and sensitivity analyses
**Discussion**		
Key results	18	Summarise key results with reference to study objectives
Limitations	19	Discuss limitations of the study, taking into account sources of potential bias or imprecision. Discuss both direction and magnitude of any potential bias
Interpretation	20	Give a cautious overall interpretation of results considering objectives, limitations, multiplicity of analyses, results from similar studies, and other relevant evidence
Generalisability	21	Discuss the generalisability (external validity) of the study results
**Other information**		
Funding	22	Give the source of funding and the role of the funders for the present study and, if applicable, for the original study on which the present article is based

**Table A3:** STROBE Analysis.

	STROBE Item Number																						COR/ Quality
	1	2	3	4	5	6	7	8	9	10	11	12	13	14	15	16	17	18	19	20	21	22	
Aneys et al. (2018)	✓	✓		✓	✓			✓	✓		✓	✓	✓	✓	✓	✓	✓	✓	✓	✓	✓	✓	18/22 (82%) High
Carvalho et al. (2019)	✓	✓	✓	✓	✓			✓	✓		✓	✓	✓	✓	✓			✓	✓	✓	✓	✓	17/22 (81%) High
Díaz et al. (2021)	✓	✓		✓	✓			✓			✓	✓	✓	✓	✓	✓	✓	✓	✓	✓	✓	✓	17/22 (77%) High
Eshetie et al. (2020)	✓	✓		✓	✓			✓			✓	✓	✓	✓	✓		✓	✓	✓	✓	✓	✓	16/22 (73%) Moderate
García-Caballero el al (2018)	✓	✓		✓	✓			✓			✓	✓	✓	✓	✓		✓	✓	✓	✓	✓	✓	15/22 (68%) Moderate
Gaubert et al. (2019)	✓	✓	✓	✓	✓			✓			✓	✓	✓	✓	✓	✓	✓	✓	✓	✓	✓	✓	18/22 (82%) High
Gutiérrez- Valencia et al. (2018)	✓	✓		✓	✓			✓			✓	✓	✓	✓	✓		✓	✓	✓	✓	✓	✓	15/22 (68%) Moderate
Liew et al. (2019)	✓	✓		✓	✓			✓			✓	✓	✓	✓	✓	✓	✓	✓	✓	✓	✓	✓	17/22 (77%) High
Monteiro et al. (2020)	✓	✓		✓	✓			✓			✓	✓	✓	✓	✓		✓	✓	✓	✓	✓		15/22 (68%) Moderate
Nieves-Pérez et al. (2018)	✓	✓		✓	✓			✓			✓		✓	✓	✓		✓	✓	✓	✓	✓	✓	15/22 (68%) Moderate
Perulero et al. (2016)	✓	✓		✓	✓			✓			✓	✓	✓	✓	✓		✓	✓	✓	✓	✓		15/22 (68%) Moderate
Stojanovic et al. (2020)	✓	✓		✓	✓			✓	✓		✓	✓	✓	✓	✓	✓	✓	✓	✓	✓	✓	✓	18/22 (82%) High

Check symbols indicate the presence of these items in the chosen studies. Blank spaces indicate the absence of the item. The evidence column indicates the number of STROBE items present in the article relative to the total number of STROBE items. The quality of studies was measured according to the Completeness of Reporting (COR) score: “low” (COR: 0–49%), “moderate” (COR: 50–74%) and “high” if ≥75% of items were met.

**Table A4:** GRADE analysis for clinical studies.

Quality Assessment						Nº of Patients		Effect		Quality
Nº of Studies	Design	Risk of Bias	Inconsistency	Indirectness	Imprecision	Intervention	Control	Relative (95%CI)	Absolute	
Strauven (2019). Interdisciplinary case conferences for nursing home staff vs. usual care										
**1**	CRCT	Serious risk of bias NHs that applied freely were included and high number of missing data)	No Serious inconsistency	No Serious indirectness	Serious imprecision (very wide range of results)	847	957	Effect in favor of the intervention: (odds ratio 1.479 [95% CI 1.062–2.059, *P* = 0.021]).		Moderate +++/++++

Abbreviations: CI: confidence interval; GRADE: Grading of Recommendations; CRCT: cluster-randomized controlled trial; NH: nursing homes; Quality of evidence grades: high (++++), moderate (+++), low (++), very low (+).

## References

[1] Organización Mundial de la Salud (2015). Informe Mundial Sobre El Envejecimiento y La Salud.

[2] Salech F., Daniel Palma Q.F., Pablo Garrido Q.F. (2016). Epidemiología del uso de medicamentos en el adulto mayor. Rev. Médica Clínica Las Condes.

[3] Pérez Díaz J., Abellán García A., Aceituno Nieto P., Ramiro Fariñas D. (2020). Un Perfil de Las Personas Mayores En España, 2020. Indicadores Estadísticos Básicos.

[4] Bo M., Gibello M., Brunetti E., Boietti E., Sappa M., Falcone Y., Aurucci M.L., Iacovino M., Fonte G., Cappa G. (2019). Prevalence and Predictors of Inappropriate Prescribing According to the Screening Tool of Older People’s Prescriptions and Screening Tool to Alert to Right Treatment Version 2 Criteria in Older Patients Discharged from Geriatric and Internal Medicine Ward. Geriatr. Gerontol. Int..

[5] De G., Lozano Montoya D.I. (2015). Buena Práctica Clínica En GERIATRÍA Presidente de La Sociedad Española de Geriatría y Gerontología (SEGG).

[6] Parodi López N., Villán Villán Y.F., Granados Menéndez M.I., Royuela A. (2014). Prescripción Potencialmente Inapropiada En Mayores de 65 Años En Un Centro de Salud de Atención Primaria. Atención Primaria.

[7] O’Sullivan D.P., O’Mahony D., Parsons C., Hughes C., Murphy K., Patterson S., Byrne S. (2013). A Prevalence Study of Potentially Inappropriate Prescribing in Irish Long-Term Care Residents. Drugs Aging.

[8] Riordan D.O., Aubert C.E., Walsh K.A., Van Dorland A., Rodondi N., Du Puy R.S., Poortvliet R.K.E., Gussekloo J., Sinnott C., Byrne S. (2018). Prevalence of Potentially Inappropriate Prescribing in a Subpopulation of Older European Clinical Trial Participants: A Cross-Sectional Study. BMJ Open.

[9] Hill-Taylor B., Sketris I., Hayden J., Byrne S., O’Sullivan D., Christie R. (2013). Application of the STOPP/START Criteria: A Systematic Review of the Prevalence of Potentially Inappropriate Prescribing in Older Adults, and Evidence of Clinical, Humanistic and Economic Impact. J. Clin. Pharm. Ther..

[10] Delgado Silveira E., Muñoz García M., Montero Errasquin B., Sánchez Castellano C., Gallagher P.F., Cruz-Jentoft A.J. (2009). Prescripción Inapropiada de Medicamentos En Los Pacientes Mayores: Los Criterios STOPP/START. Rev. Esp. Geriatr. Gerontol..

[11] Gallo C., Vilosio J., Saimovici J. (2015). Actualización de Los Criterios STOPP-START: Una Herramienta Para La Detección de Medicación Potencialmente Inadecuada En Ancianos New Version of STOPP-START Criteria: Tools for the Detection of Potentially Inappropriate Medications in the Elderly. Evidencia. Actual. En La Práctica Ambulatoria.

[12] Tommelein E., Mehuys E., Petrovic M., Somers A., Colin P., Boussery K. (2015). Potentially Inappropriate Prescribing in Community-Dwelling Older People across Europe: A Systematic Literature Review. Eur. J. Clin. Pharmacol..

[13] Storms H., Marquet K., Aertgeerts B., Claes N. (2017). Prevalence of Inappropriate Medication Use in Residential Long-Term Care Facilities for the Elderly: A Systematic Review. Eur. J. Gen. Pract..

[14] Morin L., Laroche M.L., Texier G., Johnell K. (2016). Prevalence of Potentially Inappropriate Medication Use in Older Adults Living in Nursing Homes: A Systematic Review. J. Am. Med. Dir. Assoc..

[15] Ghasemi H., Darvishi N., Salari N., Hosseinian-Far A., Akbari H., Mohammadi M. (2022). Global Prevalence of Polypharmacy among the COVID-19 Patients: A Comprehensive Systematic Review and Meta-Analysis of Observational Studies. Trop. Med. Health.

[16] Liew T.M., Lee C.S., Shawn K.L.G., Chang Z.Y. (2019). Potentially Inappropriate Prescribing Among Older Persons: A Meta-Analysis of Observational Studies. Ann. Fam. Med..

[17] Puche Cañas E., Luna del Castillo J.D. (2007). Reacciones adversas a medicamentos en pacientes que acudieron a un hospital general: Un meta-análisis de resultados. Ann. Med. Interna.

[18] Cahir C., Fahey T., Teeling M., Teljeur C., Feely J., Bennett K. (2010). Potentially Inappropriate Prescribing and Cost Outcomes for Older People: A National Population Study. Br. J. Clin. Pharmacol..

[19] Moriarty F., Hardy C., Bennett K., Smith S.M., Fahey T. (2015). Trends and Interaction of Polypharmacy and Potentially Inappropriate Prescribing in Primary Care over 15 Years in Ireland: A Repeated Cross-Sectional Study. BMJ Open.

[20] Beers M.H., Ouslander J.G., Rollingher I., Reuben D.B., Brooks J., Beck J.C. (1991). Explicit Criteria for Determining Inappropriate Medication Use in Nursing Home Residents. Arch. Intern. Med..

[21] Gallagher P., Ryan C., Byrne S., Kennedy J., O Mahony D. (2008). STOPP (Screening Tool of Older Person’s Prescriptions) and START (Screening Tool to Alert Doctors to Right Treatment). Consensus Validation. Int. J. Clin. Pharmacol. Ther..

[22] Bahat G., Bay I., Tufan A., Tufan F., Kilic C., Karan M.A. (2017). Prevalence of Potentially Inappropriate Prescribing among Older Adults: A Comparison of the Beers 2012 and Screening Tool of Older Person’s Prescriptions Criteria Version 2. Geriatr. Gerontol. Int..

[23] Ubeda A., Ferrándiz L., Maicas N., Gomez C., Bonet M., Peris J.E. (2012). Potentially Inappropriate Prescribing in Institutionalised Older Patients in Spain: The STOPP-START Criteria Compared with the Beers Criteria. Pharm. Pract..

[24] Reeve E., Shakib S., Hendrix I., Roberts M.S., Wiese M.D. (2014). The Benefits and Harms of Deprescribing. Med. J. Aust..

[25] Frankenthal D., Lerman Y., Kalendaryev E., Lerman Y. (2014). Intervention with the Screening Tool of Older Persons Potentially Inappropriate Prescriptions/Screening Tool to Alert Doctors to Right Treatment Criteria in Elderly Residents of a Chronic Geriatric Facility: A Randomized Clinical Trial. J. Am. Geriatr. Soc..

[26] Khodyakov D., Ochoa A., Olivieri-Mui B.L., Bouwmeester C., Zarowitz B.J., Patel M., Ching D., Briesacher B. (2017). STOPP/START Medication Criteria Modified for US Nursing Home Setting. J. Am. Geriatr. Soc..

[27] O’Mahony D., O’Sullivan D., Byrne S., O’Connor M.N., Ryan C., Gallagher P. (2014). STOPP/START Criteria for Potentially Inappropriate Prescribing in Older People: Version 2. Age Ageing.

[28] Delgado Silveira E., Montero Errasquín B., Muñoz García M., Vélez-Díaz-Pallarés M., Lozano Montoya I., Sánchez-Castellano C., Cruz-Jentoft A.J. (2015). Mejorando La Prescripción de Medicamentos En Las Personas Mayores: Una Nueva Edición de Los Criterios STOPP-START. Rev. Esp. Geriatr. Gerontol..

[29] Moriarty F., Bennett K., Cahir C., Kenny R.A., Fahey T. (2016). Potentially Inappropriate Prescribing According to STOPP and START and Adverse Outcomes in Community-Dwelling Older People: A Prospective Cohort Study. Br. J. Clin. Pharmacol..

[30] Sevilla-Sánchez D., Espaulella-Panicot J., de Andrés-Lazaro A.M., Torres-Allezpuz R., Soldevila-Llagostera M., Codina-Jane C. (2012). Medicación Potencialmente Inapropiada Al Ingreso En Una Unidad de Media Estancia Según Los Criterios STOPP & START. Rev. Esp. Geriatr. Gerontol..

[31] Cruz-Esteve I., Marsal-Mora J.R., Galindo-Ortego G., Galván-Santiago L., Serrano-Godoy M., Ribes-Murillo E., Real-Gatius J. (2016). Análisis Poblacional de La Prescripción Potencialmente Inadecuada En Ancianos Según Criterios STOPP/START (Estudio STARTREC). Atención Primaria.

[32] San-José A., Agustí A., Vidal X., Formiga F., Gómez-Hernández M., García J., López-Soto A., Ramírez-Duque N., Torres O.H., Barbé J. (2015). Inappropriate Prescribing to the Oldest Old Patients Admitted to Hospital: Prevalence, Most Frequently Used Medicines, and Associated Factors. BMC Geriatr..

[33] Tosato M., Landi F., Martone A.M., Cherubini A., Corsonello A., Volpato S., Bernabei R., Onder G. (2014). Potentially Inappropriate Drug Use among Hospitalised Older Adults: Results from the CRIME Study. Age Ageing.

[34] Fialová D., Laffon B., Marinković V., Tasić L., Doro P., Sόos G., Mota J., Dogan S., Brkić J., Teixeira J.P. (2019). Medication Use in Older Patients and Age-Blind Approach: Narrative Literature Review (Insufficient Evidence on the Efficacy and Safety of Drugs in Older Age, Frequent Use of PIMs and Polypharmacy, and Underuse of Highly Beneficial Nonpharmacological Strategies). Eur. J. Clin. Pharmacol..

[35] Hill-Taylor B., Walsh K.A., Stewart S., Hayden J., Byrne S., Sketris I.S. (2016). Effectiveness of the STOPP/START (Screening Tool of Older Persons’ Potentially Inappropriate Prescriptions/Screening Tool to Alert Doctors to the Right Treatment) Criteria: Systematic Review and Meta-Analysis of Randomized Controlled Studies. J. Clin. Pharm. Ther..

[36] Tian F., Chen Z., Zhou D., Mo L. (2022). Prevalence of Polypharmacy and Potentially Inappropriate Medication Use in Older Lung Cancer Patients: A Systematic Review and Meta-Analysis. Front. Pharmacol..

[37] Atmaja D.S., Yulistiani, Suharjono, Zairina E. (2022). Detection Tools for Prediction and Identification of Adverse Drug Reactions in Older Patients: A Systematic Review and Meta-Analysis. Sci. Rep..

[38] Lee J.W., Li M., Boyd C.M., Green A.R., Szanton S.L. (2022). Preoperative Deprescribing for Medical Optimization of Older Adults Undergoing Surgery: A Systematic Review. J. Am. Med. Dir. Assoc..

[39] Alshammari H., Al-Saeed E., Ahmed Z., Aslanpour Z. (2021). Reviewing Potentially Inappropriate Medication in Hospitalized Patients Over 65 Using Explicit Criteria: A Systematic Literature Review. Drug. Healthc. Patient Saf..

[40] Mekonnen A.B., Redley B., de Courten B., Manias E. (2021). Potentially Inappropriate Prescribing and Its Associations with Health-Related and System-Related Outcomes in Hospitalised Older Adults: A Systematic Review and Meta-Analysis. Br. J. Clin. Pharmacol..

[41] Farhat A., Al-Hajje A., Csajka C., Panchaud A. (2021). Clinical and Economic Impacts of Explicit Tools Detecting Prescribing Errors: A Systematic Review. J. Clin. Pharm. Ther..

[42] Thomas R.E., Thomas B.C. (2019). A Systematic Review of Studies of the STOPP/START 2015 and American Geriatric Society Beers 2015 Criteria in Patients ≥ 65 Years. Curr. Aging Sci..

[43] Cooper J.A., Cadogan C.A., Patterson S.M., Kerse N., Bradley M.C., Ryan C., Hughes C.M. (2015). Interventions to Improve the Appropriate Use of Polypharmacy in Older People: A Cochrane Systematic Review. BMJ Open.

[44] Liberati A., Altman D.G., Tetzlaff J., Mulrow C., Gøtzsche P.C., Ioannidis J.P.A., Clarke M., Devereaux P.J., Kleijnen J., Moher D. (2009). The PRISMA Statement for Reporting Systematic Reviews and Meta-Analyses of Studies That Evaluate Healthcare Interventions: Explanation and Elaboration. BMJ.

[45] Page M.J., McKenzie J.E., Bossuyt P.M., Boutron I., Hoffmann T.C., Mulrow C.D., Shamseer L., Tetzlaff J.M., Akl E.A., Brennan S.E. (2021). The PRISMA 2020 Statement: An Updated Guideline for Reporting Systematic Reviews. BMJ.

[46] Von Elm E., Altman D.G., Egger M., Pocock S.J., Gøtzsche P.C., Vandenbroucke J.P. (2007). The Strengthening the Reporting of Observational Studies in Epidemiology (STROBE) Statement: Guidelines for Reporting Observational Studies. Ann. Intern. Med..

[47] Suvarna B., Suvarna A., Phillips R., Juster R.-P., McDermott B., Sarnyai Z. (2020). Health Risk Behaviours and Allostatic Load: A Systematic Review. Neurosci. Biobehav. Rev..

[48] Osei F., Block A., Wippert P.-M., Qorbani M., Djalalinia S., Tabatabaei-Malazy O. (2022). Association of Primary Allostatic Load Mediators and Metabolic Syndrome (MetS): A Systematic Review. Front. Endocrinol..

[49] Guyatt G., Oxman A.D., Akl E.A., Kunz R., Vist G., Brozek J., Norris S., Falck-Ytter Y., Glasziou P., Debeer H. (2011). GRADE Guidelines: 1. Introduction-GRADE Evidence Profiles and Summary of Findings Tables. J. Clin. Epidemiol..

[50] Díaz Planelles I., Saurí Ferrer I., Trillo-Mata J.L., Navarro-Pérez J. (2021). Analysis of Potentially Inappropriate Prescriptions According to the START Criteria in Nursing Homes. Rev. Esp. Geriatr. Gerontol..

[51] Gutiérrez-Valencia M., Izquierdo M., Lacalle-Fabo E., Marín-Epelde I., Ramón-Espinoza M.F., Domene-Domene T., Casas-Herrero Á., Galbete A., Martínez-Velilla N. (2018). Relationship between Frailty, Polypharmacy, and Underprescription in Older Adults Living in Nursing Homes. Eur. J. Clin. Pharmacol..

[52] García-Caballero T.M., Lojo J., Menéndez C., Fernández-Álvarez R., Mateos R., Garcia-Caballero A. (2018). Polimedication: Applicability of a Computer Tool to Reduce Polypharmacy in Nursing Homes. Int. Psychogeriatr..

[53] Perulero M.L.M. (2016). Impacto de un programa de prescripción prudente en residentes de centros sociosanitarios. Pharm. Care Esp..

[54] Carvalho R., Lavrador M., Cabral A.C., Veríssimo M.T., Figueiredo I.V., Fernandez-Llimos F., Margarida Castel-Branco M. (2019). Patients’ Clinical Information Requirements to Apply the STOPP/START Criteria. Int. J. Clin. Pharm..

[55] Monteiro C., Canário C., Ribeiro M.Â., Duarte A.P., Alves G. (2020). Medication Evaluation in Portuguese Elderly Patients According to Beers, STOPP/START Criteria and EU(7)-PIM List—An Exploratory Study. Patient Prefer. Adherence.

[56] Anrys P.M.S., Strauven G.C., Foulon V., Degryse J.M., Henrard S., Spinewine A. (2018). Potentially Inappropriate Prescribing in Belgian Nursing Homes: Prevalence and Associated Factors. J. Am. Med. Dir. Assoc..

[57] Strauven G., Anrys P., Vandael E., Henrard S., De Lepeleire J., Spinewine A., Foulon V. (2019). Cluster-Controlled Trial of an Intervention to Improve Prescribing in Nursing Homes Study. J. Am. Med. Dir. Assoc..

[58] Stojanović M., Vuković M., Jovanović M., Dimitrijević S., Radenković M. (2019). GheOP^3^S Tool and START/STOPP Criteria Version 2 for Screening of Potentially Inappropriate Medications and Omissions in Nursing Home Residents. J. Eval. Clin. Pract..

[59] Gaubert-Dahan M.-L., Sebouai A., Tourid W., Fauvelle F., Aikpa R., Bonnet-Zamponi D. (2019). The Impact of Medication Review with Version 2 STOPP (Screening Tool of Older Person’s Prescriptions) and START (Screening Tool to Alert Doctors to Right Treatment) Criteria in a French Nursing Home: A 3-Month Follow-up Study. Ther. Adv. Drug Saf..

[60] Nieves-Pérez B.F., Hostos S.G.-D., Frontera-Hernández M.I., González I.C., Muñoz J.J.H. (2018). Potentially Inappropriate Medication Use Among Institutionalized Older Adults at Nursing Homes in Puerto Rico. Consult. Pharm..

[61] Eshetie T.C., Roberts G., Nguyen T.A., Gillam M.H., Maher D., Ellett L.M.K. (2020). Potentially Inappropriate Medication Use and Related Hospital Admissions in Aged Care Residents: The Impact of Dementia. Br. J. Clin. Pharmacol..

[62] Liew N.Y., Chong Y.Y., Yeow S.H., Kua K.P., Saw P.S., Lee S.W.H. (2019). Prevalence of Potentially Inappropriate Medications among Geriatric Residents in Nursing Care Homes in Malaysia: A Cross-Sectional Study. Int. J. Clin. Pharm..

[63] Anrys P., Strauven G., Boland B., Dalleur O., Declercq A., Degryse J.-M., De Lepeleire J., Henrard S., Lacour V., Simoens S. (2016). Collaborative Approach to Optimise MEdication Use for Older People in Nursing Homes (COME-ON): Study Protocol of a Cluster Controlled Trial. Implement. Sci..

[64] O’Mahony D. (2020). STOPP/START Criteria for Potentially Inappropriate Medications/Potential Prescribing Omissions in Older People: Origin and Progress. Expert Rev. Clin. Pharmacol..

[65] Hernandez Martin J., Merino-Sanjuán V., Peris-Martí J., Correa-Ballester M., Vial-Escolano R., Merino-Sanjuán M. (2018). Applicability of the STOPP/START Criteria to Older Polypathological Patients in a Long-Term Care Hospital. Eur. J. Hosp. Pharm..

[66] Díez R., Cadenas R., Susperregui J., Sahagún A.M., Fernández N., García J.J., Sierra M., López C. (2022). Drug-Related Problems and Polypharmacy in Nursing Home Residents: A Cross-Sectional Study. Int. J. Environ. Res. Public Health.

[67] Mills K.T., Stefanescu A., He J. (2020). The Global Epidemiology of Hypertension. Nat. Rev. Nephrol..

[68] Inzitari M., Merino Méndez R., Santaeugenia S., Pascual Arce B., Montero Leno A., Sunyer B., López S.A.C. Prescripción Potencialmente Inadecuadas En Dos Centros Sociosanitarios Según Los Criterios START-STOPP. https://aquas.gencat.cat/web/.content/minisite/aquas/publicacions/2017/prescripcion_sociosanitarios_START-STOPP_Red_aquas2017.pdf.

[69] Primejdie D.P., Bojita M.T., Popa A. (2016). Potentially Inappropriate Medications in Elderly Ambulatory and Institutionalized Patients: An Observational Study. BMC Pharmacol. Toxicol..

[70] Awad A., Hanna O. (2019). Potentially Inappropriate Medication Use among Geriatric Patients in Primary Care Setting: A Cross-Sectional Study Using the Beers, STOPP, FORTA and MAI Criteria. PLoS ONE.

[71] Rivas-Cobas P.C., Ramírez-Duque N., Gómez Hernández M., García J., Agustí A., Vidal X., Formiga F., López-Soto A., Torres O.H., San-José A. (2017). Características Del Uso Inadecuado de Medicamentos En Pacientes Pluripatológicos de Edad Avanzada. Gac. Sanit..

[72] Blanco-Reina E., Ariza-Zafra G., Ocaña-Riola R., León-Ortiz M. (2014). 2012 American Geriatrics Society Beers Criteria: Enhanced Applicability for Detecting Potentially Inappropriate Medications in European Older Adults? A Comparison with the Screening Tool of Older Person’s Potentially Inappropriate Prescriptions. J. Am. Geriatr. Soc..

[73] Ruiz-Millo O., Climente-Martí M., Navarro-Sanz J.R. (2018). Improvement on Prescribing Appropriateness after Implementing an Interdisciplinary Pharmacotherapy Quality Programme in a Long-Term Care Hospital. Eur. J. Hosp. Pharm..

[74] Renom-Guiteras A., Thürmann P.A., Miralles R., Klaaßen-Mielke R., Thiem U., Stephan A., Bleijlevens M.H.C., Jolley D., Leino-Kilpi H., Hallberg I.R. (2018). Potentially Inappropriate Medication among People with Dementia in Eight European Countries. Age Ageing.

[75] Lind K.E., Raban M.Z., Georgiou A., Westbrook J.I. (2019). NSAID Use among Residents in 68 Residential Aged Care Facilities 2014 to 2017: An Analysis of Duration, Concomitant Medication Use, and High-Risk Conditions. Pharmacoepidemiol. Drug Saf..

[76] Wright D.J., Maskrey V., Blyth A., Norris N., Alldred D.P., Bond C.M., Desborough J., Hughes C.M., Holland R.C. (2020). Systematic Review and Narrative Synthesis of Pharmacist Provided Medicines Optimisation Services in Care Homes for Older People to Inform the Development of a Generic Training or Accreditation Process. Int. J. Pharm. Pract..

